# The prognostic value of triglyceride glucose–waist height ratio index in patients with heart failure after acute myocardial infarction: a retrospective study

**DOI:** 10.3389/fcvm.2026.1762185

**Published:** 2026-08-12

**Authors:** Hongru Fan, Qingjun Jiang, Liqun He

**Affiliations:** 1 Department of Cardiology, Wuhan No.1 Hospital, Wuhan, Hubei, China; 2 Department of Vascular & Endovascular Surgery, Changzheng Hospital, Naval Medical University, Shanghai, China

**Keywords:** acute myocardial infarction, heart failure, prognosis, restricted cubic splines, TyG-WHtR

## Abstract

**Background:**

Heart failure (HF) after acute myocardial infarction (AMI) remains a major clinical challenge. The triglyceride glucose-waist-to-height ratio (TyG-WHtR) integrates metabolic and obesity indices, yet its prognostic value for AMI-HF is unclear.

**Methods:**

This retrospective study enrolled 738 AMI patients undergoing PCI from January 2019 to June 2024. Baseline TyG-WHtR was calculated pre-discharge. The primary outcome was HF occurrence within 1-year follow-up. Multivariate logistic regression and restricted cubic splines (RCS) assessed associations. Receiver operating characteristic (ROC) curves and DeLong tests compared TyG-WHtR against the GRACE score and other metabolic indices. Subgroup analyses were performed based on AMI-to-PCI time and ESC-defined HF classifications.

**Results:**

A total of 136 patients (18.4%) developed HF. TyG-WHtR was an independent predictor of AMI-HF (adjusted OR = 1.192, 95% CI: 1.021∼1.392, *p* = 0.026), persisting after adjusting for the post-PCI GRACE score. RCS revealed a positive nonlinear dose-response relationship (p-nonlinear=0.034). TyG-WHtR demonstrated good discrimination (AUC=0.769, 95% CI: 0.726∼0.811), outperforming TyG-WC and TyG-BMI (both *p* < 0.05). Notably, TyG-WHtR showed superior predictive value in HFpEF (AUC=0.815) compared to GRACE (AUC=0.761, *p* = 0.012), whereas GRACE performed better in HFrEF. The predictive value was consistent across AMI-to-PCI time strata (p-interaction=0.584). Baseline TyG-WHtR correlated inversely with 1-year LVEF (r = −0.327, *p* < 0.001) in HF patients.

**Conclusion:**

TyG-WHtR is an independent risk factor for 1-year AMI-HF, particularly in HFpEF phenotypes. It provides incremental prognostic value beyond the GRACE score and may guide early risk stratification in post-PCI AMI patients.

## 1 Introduction

Cardiovascular disease (CVD) has emerged as the leading cause of global morbidity, with heart failure (HF) posing a significant burden on healthcare systems worldwide because of its high incidence and mortality rates (1, 2). HF is the terminal event in the deterioration of various cardiac diseases, with acute myocardial infarction (AMI)-induced ischemic myocardial necrosis being a significant cause of HF. Studies have shown that among AMI patients undergoing percutaneous coronary intervention (PCI), the postoperative incidence of HF still reaches 36% (3). Although the management of HF after AMI has gradually improved in recent years, the reasons why HF patients continue to deteriorate despite aggressive treatment remain unclear. There is an urgent need for a novel and convenient method to rapidly identify HF risk, assisting clinical physicians in risk stratification and early diagnosis, thereby improving patient prognosis (4).

The triglyceride‒glucose index (TyG) is a commonly used indicator for assessing insulin resistance and metabolic abnormalities (5). Studies have shown that the TyG index is significantly correlated with cardiovascular disease (CVD) and has excellent performance in predicting the occurrence and prognosis of CVD, such as myocardial infarction and stroke (6, 7). Obesity is a significant factor contributing to various diseases. Research has indicated that central obesity can induce cardiovascular disease (CVD) through multiple pathophysiological mechanisms (8). The waist-to-height ratio (WHtR) has become an internationally recognized simple indicator for assessing central obesity. Consequently, the combination of TyG and WHtR to form the triglyceride‒glucose‒waist‒to‒height ratio (TyG‒WHtR) has gained increasing attention. Two recent large-scale studies revealed that, compared with the TyG index, the TyG-WHtR index demonstrated superior performance in identifying high-risk populations for CVD events and CVD mortality while confirming the independent association between the TyG-WHtR index and CVD event risk (9, 10). However, the ability of the TyG-WHtR index to predict heart failure remains unknown, particularly in populations with acute myocardial infarction complicated by heart failure, where no studies have been conducted.

This study investigated the association between the TyG-WHtR index and heart failure in acute myocardial infarction patients, aiming to determine its effectiveness as a predictor of post-acute myocardial infarction heart failure (AMI-HF).

## 2 Methods

### 2.1 Study subjects

A retrospective study selected patients with acute myocardial infarction (AMI) who underwent percutaneous coronary intervention (PCI) in the Department of Cardiology, Wuhan No.1 Hospital, from January 2019 to June 2024. The inclusion criteria were as follows: (1) hospitalized patients aged ≥18 years; (2) patients with a confirmed diagnosis of AMI; (3) first-time occurrence and receiving PCI treatment; and (4) postoperative follow-up duration of at least 1 year. Exclusion criteria: (1) preexisting heart failure before PCI; (2) lack of baseline echocardiogram or other missing clinical data; (3) comorbidities such as malignant tumors or valvular heart disease; and (4) loss to follow-up or incomplete records. All enrolled patients provided written informed consent before clinical data collection, and the study was approved by the hospital's Ethics Committee.

### 2.2 Baseline data collection

Basic information: Age, sex, weight, waist circumference, height, smoking history and drinking history. Medical history: Underlying diseases such as diabetes, hypertension, hyperlipidemia, etc. Blood test indicators: Total cholesterol, triglycerides (triacylglycerol), fasting blood glucose, creatinine clearance rate, N-terminal pro-B-type natriuretic peptide (NT-proBNP), time interval to PCI, and the GRACE score. The GRACE risk score at discharge was calculated for each patient by assigning the appropriate number of points for each of the 9 prognostic variables that enter into the calculation.

The primary outcome was the incidence of HF at the 1-year follow-up. HF was diagnosed based on clinical symptoms, signs, and objective evidence, including elevated NT-proBNP levels and reduced left ventricular ejection fraction (LVEF) measured at the 1-year follow-up. Postoperative NT-proBNP and LVEF values were utilized exclusively as endpoint diagnostic criteria and were strictly excluded from the baseline predictive models to avoid circular reasoning.

### 2.3 Definition of heart failure after myocardial infraction

The endpoint event was acute myocardial infarction with heart failure (AMI-HF). The diagnosis of AMI-HF requires comprehensive assessment combining symptoms, plasma natriuretic peptide levels, and echocardiography: (1) history of myocardial infarction; (2) symptoms and/or signs of heart failure; (3) N-terminal pro-B-type natriuretic peptide (NT-proBNP) ≥ 125 pg/mL or B-type natriuretic peptide (BNP) ≥ 35 pg/mL; and (4) echocardiography showing structural and functional abnormalities. Other causes of heart failure must be excluded.

### 2.4 Calculation of the triglyceride glucose‒waist to height ratio (TyG‒WHtR)

(1) WHtR = Waist circumference (cm)/height (cm)
(2) TyG = Ln[Fasting triglycerides (mg/dL)   ×   Fasting glucose (mg/dL)/2]
(3) TyG-WHtR = TyG   ×   WHtR
(4) TyG-WC = TyG   ×   waist circumference (WC)
(5) TyG-BMI = TyG   ×   body mass index (BMI)

### 2.5 Statistical methods

Statistical analysis was performed via SPSS 24 software. Normally distributed data are expressed as the means ± standard deviations (x ± s), and comparisons between two groups were conducted via t tests, whereas comparisons among multiple groups were analyzed via analysis of variance (ANOVA). Nonnormally distributed data or data with unequal variances are presented as the median and interquartile range (M) (P25, P75), and Mann‒Whitney U tests were employed. Categorical data are described as counts and percentages (%), with comparisons between two groups performed via chi-square tests or Fisher's exact tests. A *P* value <0.05 was considered to indicate statistical significance.

To compare the predictive performance of TyG-WHtR with other obesity-related indices, we additionally calculated TyG-WC and TyG-BMI. We calculated the GRACE risk score for each patient to compare TyG-WHtR with established clinical predictors. ROC curve analyses were then conducted for GRACE score, and TyG-WHtR, and the corresponding AUCs were compared using DeLong's test. To assess the independent predictive value of TyG-WHtR, we constructed two nested logistic regression models. Model 1 was adjusted for age, sex, and time from AMI to PCI. Model 2 was further adjusted for smoking, diabetes, dyslipidemia, hypertension, and the post-PCI GRACE score.

## 3 Results

### 3.1 Baseline characteristics

**Figure 1:**
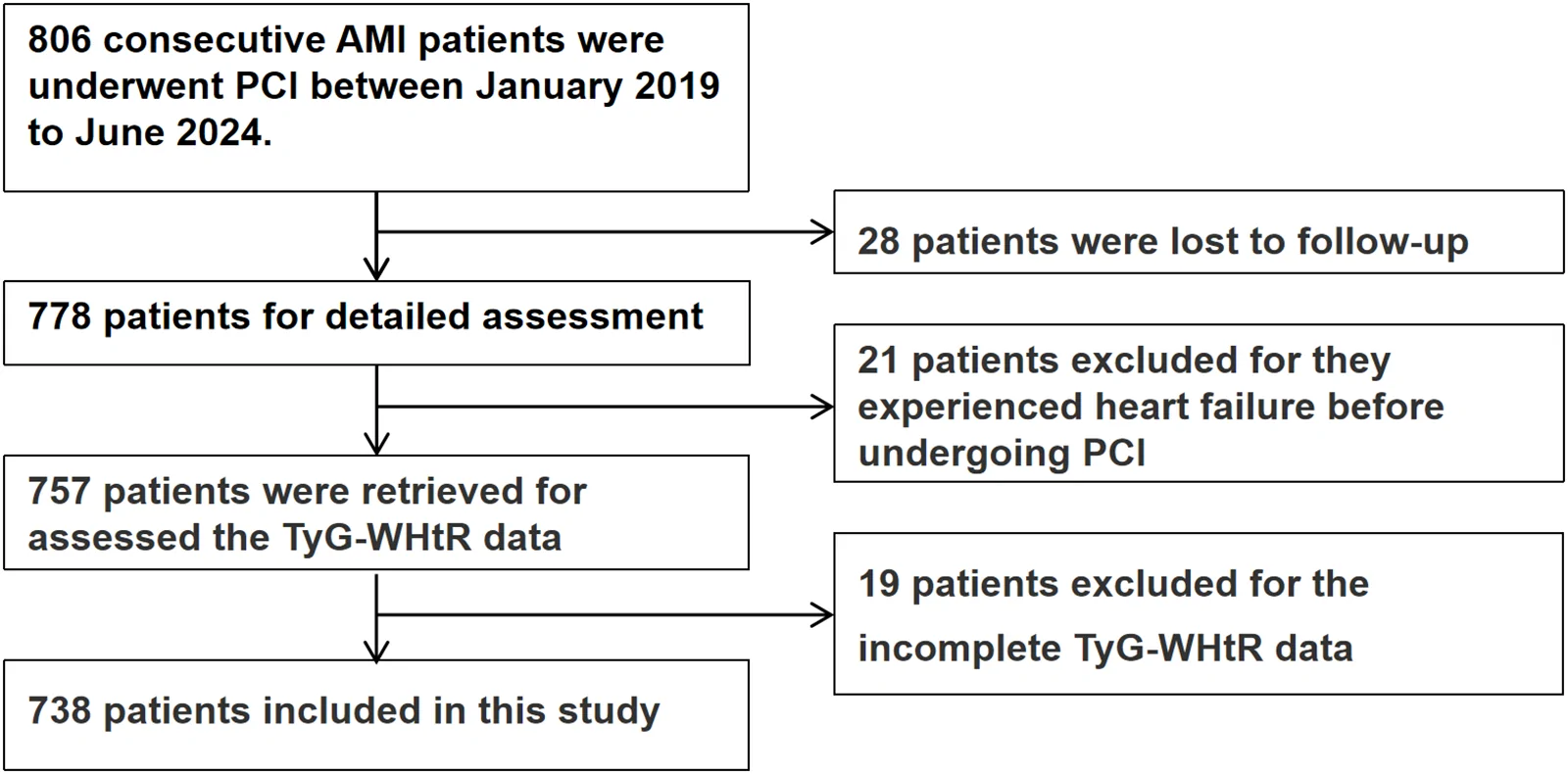
Flow chart of the study population.

A total of 806 patients with AMI were screened, and 738 met the inclusion criteria (Figure 1). The baseline characteristics of the study population are detailed in Table 1. Among the 738 enrolled patients, 136 (18.4%) developed heart failure within one year after surgery. The results revealed that the AMI-HF group had an average age of 63.7 ± 5.9 years, with 55.1% males, whereas the post-AMI normal group had an average age of 58.3 ± 5.3 years, with 53.7% males. The TyG-WHtR index in the heart failure group was significantly greater than that in the nonheart failure group (5.09 ± 0.38 vs. 5.01 ± 0.386, *P* < 0.001). Statistically significant differences in age, smoking status, diabetes status, lipid levels, waist circumference, and other indicators were detected between the two groups, whereas no significant differences in sex, height, or the TyG index were detected.

**Table 1:** Baseline characteristics of AMI patients with and without heart failure.

Index	Heart Failure	Non-Heart Failure	*P* value
Number of patients	136	602	
Age/years	63.72 ± 5.91	58.32 ± 5.31	<0.001
Male, n(%)	75 (55.14)	323 (53.65)	0.752
Height/cm	168.3 ± 6.4	168.35 ± 4.71	0.621
Waist circumference/cm	90.38 ± 4.97	89.46 ± 5.87	0.015
WHtR	0.54 ± 0.03	0.53 ± 0.03	0.060
Smoking, n(%)	77 (56.61)	241 (40.03)	<0.001
Diabetes, n(%)	62 (45.59)	150 (24.92)	<0.001
Hypertension, n(%)	88 (64.70)	355 (58.97)	0.217
Dyslipidemia, n	98 (72.06)	310 (51.50)	<0.001
TC/(mg•dL−1)	153.11 ± 39.93	153.47 ± 31.08	0.454
TG/(mg•dL−1)	247.46 ± 75.96	233.05 ± 51.27	0.023
FBG/(mg•dL−1)	117.04 ± 36.26	115.36 ± 36.87	0.630
LVEF(%)	67.2 ± 10.1	68.7 ± 8.7	0.272
NT-proBNP/(pg•mL-1)	106.2 (83.5, 126.9)	102.3 (81.2, 120.4)	0.177
TyG	9.48 ± 0.45	9.44 ± 0.38	0.147
TyG-WHtR	5.09 ± 0.38	5.01 ± 0.386	0.030
Time from AMI to PCI (h)	7.21 ± 3.90	4.78 ± 3.71	<0.001
One-year follow-up*			
LVEF(%)	41.3 ± 12.1	66.9 ± 10.2	<0.001
NT-proBNP/(pg•mL^−1^)	2,769.5 (1,523.2, 4,366.7)	82.9 (63.5, 123.7)	<0.001

TC, total cholesterol; TG, triglyceride; FBG, fasting blood glucose; TyG-WHtR, triglyceride glucose-waist-to-height ratio.

* Baseline LVEF and NT-proBNP were measured pre-discharge after PCI. The follow-up LVEF and NT-proBNP (shown in the lower section) were obtained at 1 year and used solely for the diagnosis of the endpoint event (heart failure).

### 3.2 Univariate and multivariate logistic regression analysis of the TyG-WHtR index and AMI-HF

As shown in Table 2, we evaluated baseline predictors of 1-year post-PCI heart failure. All variables were collected pre-discharge after PCI; NT-proBNP and LVEF were used only for endpoint diagnosis and excluded from models. Univariate analysis linked TyG-WHtR, post-PCI GRACE score, age, male sex, longer PCI delay, smoking, diabetes, dyslipidemia to elevated heart failure risk.

**Table 2:** Results of univariate and multivariate logistic regression analyses for predicting AMI-HF at 1-year follow-up.

Factors	Univariate logistic regression		Model 1: Multivariate logistic regression		Model 2: Multivariate logistic regression	
	Odds Ratio (95% CI)	*P* value	Odds Ratio (95% CI)	*P* value	Odds Ratio (95% CI)	*P* value
TyG-WHtR	1.716 (1.051∼2.802)	0.031	1.248 1.042∼1.495)	0.016	1.192 (1.021∼1.392)	0.026
GRACE Score	1.031 (1.022∼1.040)	<0.001			1.026 (1.017∼1.035)	<0.001
Age (per year)	1.182 (1.139∼1.226)	<0.001	1.165 (1.121∼1.211)	<0.001	1.148 (1.103∼1.194)	<0.001
Male	1.346 (1.291∼1.572)	<0.001	1.188 (1.012∼1.395)	0.035	1.152 (0.992∼1.337)	0.063
Time to PCI (h)	1.228 (1.172∼1.285)	<0.001	1.179 (1.124∼1.238)	<0.001	1.156 (1.101∼1.214)	<0.001
Smoking	1.984 (1.532∼2.568)	<0.001	-	-	1.398 (1.068∼1.830)	0.015
Diabetes	2.561 (1.953∼3.358)	<0.001	-	-	1.652 (1.221∼2.236)	0.001
Dyslipidemia	2.412 (1.782∼3.265)	<0.001	-	-	1.285 (0.956∼1.727)	0.097
Hypertension	1.285 (0.982∼1.681)	0.067	-	-	1.074 (0.794∼1.452)	0.641
TC (mg/dL)	0.999 (0.992∼1.006)	0.782	-	-	0.995 (0.987∼1.003)	0.208
TG (mg/dL)	1.004 (1.001∼1.007)	0.008	-	-	-	Remove*

CI, confidence interval; TC, total cholesterol; TG, triglyceride; FBG, fasting blood glucose; TyG-WHtR, triglyceride glucose-waist-to-height ratio.

Model 1: Adjusted for age, sex, and time from AMI onset to PCI.

Model 2: Adjusted for variables in Model 1 plus smoking, diabetes, dyslipidemia, hypertension, and the post-PCI GRACE score (9-item discharge version). Variance Inflation Factor (VIF) for all variables in Model 2 ranged from 1.1 to 2.3, indicating no severe multicollinearity.

*Triglycerides (TG) were excluded from Model 2 due to multicollinearity with TyG-WHtR (VIF > 5).

In Model 1 (adjusted for age, sex, PCI delay), TyG-WHtR remained an independent risk factor [OR = 1.248, 95%CI(1.042–1.495), *P* = 0.016]. Model 2 added smoking, diabetes, dyslipidemia, hypertension and GRACE score. TG was excluded due to collinearity with TyG-WHtR; all variables had variance inflation factors <2.5, ruling out multicollinearity. GRACE was strongly predictive: each 10-point increase raised heart failure risk by 29% [OR = 1.026 per point, 95%CI (1.017∼1.035), *P* < 0.001]. Notably, TyG-WHtR retained significance after full adjustment [OR = 1.176, 95%CI(1.012∼1.367), *P* = 0.034]. Sex, dyslipidemia, hypertension and total cholesterol were no longer significant in Model 2.

### 3.3 Nonlinear analysis

**Figure 2:**
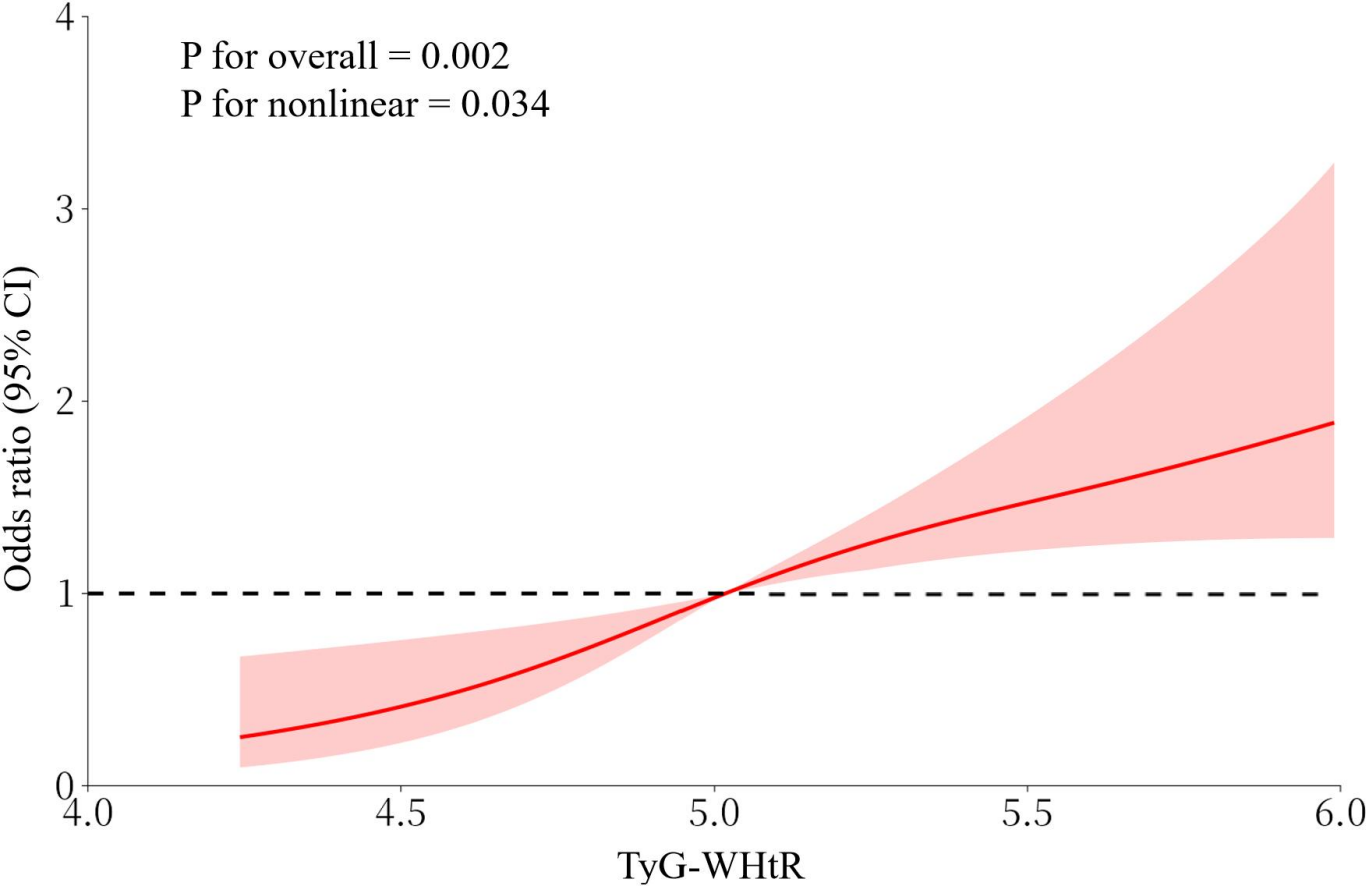
Restricted cubic spline curves between the TyG-WHtR index and AMI-HF.

Restricted cubic spline (RCS) regression models were used to further examine the association between the TyG-WHtR and the risk of AMI-HF (Figure 2). We found that there was a positive nonlinear dose‒response relationship between the TyG-WHtR index and AMI-HF (p for overall = 0.002, *p* for nonlinea*r* = 0.034). RCS regression models revealed a progressive nonlinear increase in AMI-HF risk when the TyG-WHtR index exceeded 5.01.

### 3.4 Receiver operating characteristic analysis

**Figure 3:**
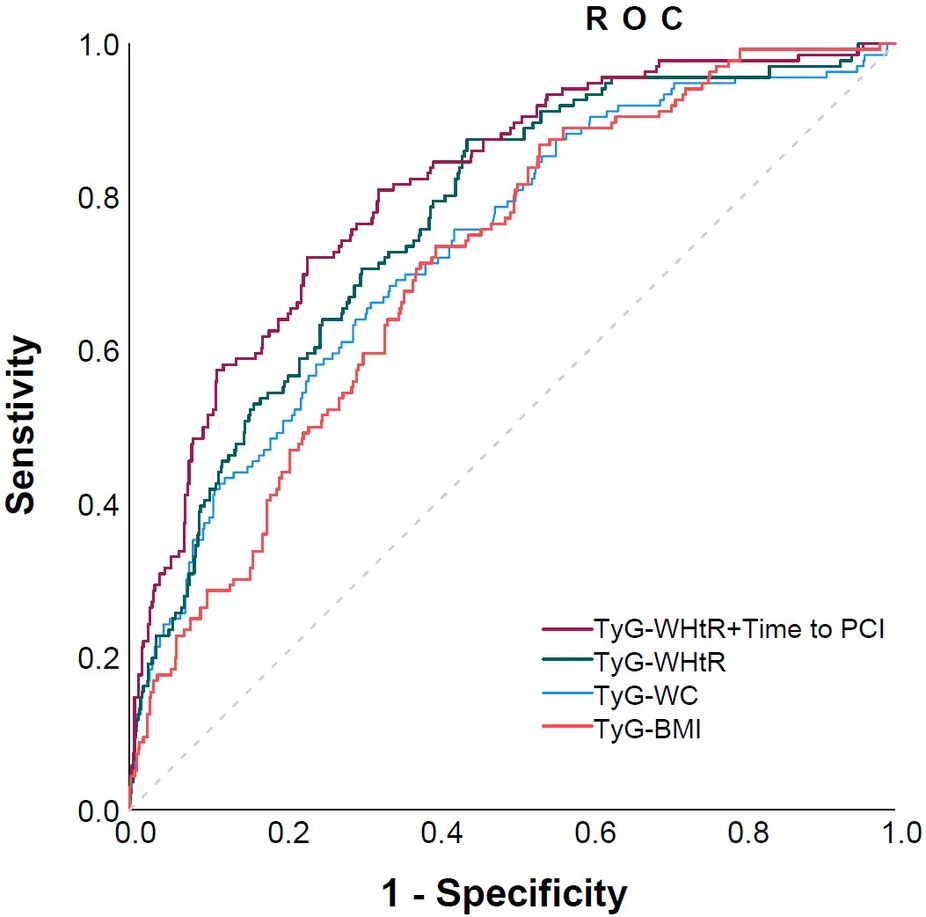
Receiver operating characteristic curves for baseline TyG-WHtR + time to PCI, TyG-WHtR, TyG-WC, and TyG-BMI predicting AMI-HF.

ROC curves were used to evaluate the predictive efficacy of the TyG-WHtR index in AMI patients with HF. As presented in the Table 3，the ROC curve indicated that the TyG-WHtR index demonstrated an AUC of 0.769 (95% CI: 0.726∼0.811), which was significantly higher than that of TyG-WC [AUC=0.733, 95% CI (0.686∼0.780), PDeLong = 0.042] and TyG-BMI [AUC=0.711, 95% CI (0.666∼0.755), PDeLong = 0.018]. When the TyG-WHtR was combined with the time interval from AMI onset to PCI, the combined model [AUC=0.811, 95% CI (0.771∼0.850), *P* < 0.001]. (Figure 3) The TyG-WHtR + PCI Time model showed adequate calibration according to the Hosmer–Lemeshow test (*χ*2 = 10.943, *P* = 0.205). This finding suggests that the TyG-WHtR is valuable for predicting the occurrence of HF when the TyG-WHtR is greater than 5.48.

**Table 3:** Comparative predictive performance of TyG-WHtR and other metabolic-obesity indices for AMI-HF.

Factors	AUC (95% CI)	Sensitivity (%)	Specificity (%)	Optimal cutoff	*P* value
TyG-WHtR	0.769 (0.726∼0.811)	87.5	56.0	5.48	Reference
TyG-WHtR + Time to PCI	0.811 (0.771∼0.850)	81.6	72.8	0.38	<0.001
TyG-WC	0.733 (0.686∼0.780)	79.4	57.1	856.3	0.042
TyG-BMI	0.711 (0.666∼0.755)	74.3	60.2	248.6	0.018

TyG-WC, TyG   ×   Waist Circumference (cm); TyG-BMI, TyG   ×   Body Mass Index (kg/m^2^). DeLong test was used to compare AUCs.

### 3.5 Subgroup and HF classification analyses

TyG-WHtR predicted 1-year HF across all AMI-to-PCI time strata (P-interaction=0.584; Table 4). When classified by LVEF per ESC 2021 criteria, TyG-WHtR showed strongest discrimination in HFpEF (AUC=0.815), significantly outperforming the GRACE score (0.761, *P* = 0.012). In contrast, GRACE performed better in HFrEF (0.791 vs. 0.748, *P* = 0.108), suggesting TyG-WHtR captures distinct metabolic risks prevalent in preserved ejection fraction phenotypes. In all 136 AMI-HF patients, baseline TyG-WHtR correlated inversely with 1-year LVEF (r = −0.327, *P* < 0.001).

**Table 4:** Subgroup analysis by AMI-to-PCI time and prognostic performance across HF classifications.

Subgroup / HF type	N (HF/ total)	TyG-WHtR OR (95%CI)	*P* value	TyG-WHtR AUC (95%CI)	GRACE AUC (95%CI)	*P* Delong
AMI-to-PCI time						
≤6 h	58/312	1.205 (1.021∼1.422)	0.027	-	-	-
6–12 h	49/248	1.188 (1.015∼1.391)	0.031	-	-	-
>12 h	29/178	1.231 (1.048∼1.446)	0.011	-	-	-
P for interaction	-	-	0.584	-	-	-
HF classification (1-year)						
HFrEF (LVEF < 40%)	62/136	1.137 (0.961∼1.348)	0.132	0.748 (0.689∼0.807)	0.791 (0.734∼0.847)	0.108
HFmrEF (40%–49%)	38/136	1.215 (1.042∼1.419)	0.013	0.779 (0.716∼0.840)	0.768 (0.705∼0.831)	0.064
HFpEF (LVEF≥50%)	36/136	1.289 (1.113∼1.494)	<0.001	0.815 (0.754∼0.872)	0.761 (0.698∼0.823)	0.012

## 4 Discussion

This study investigated the association between the TyG-WHtR index and AMI-HF. The results revealed that TyG-WHtR was significantly greater in the HF group than in the non-HF group. Multivariate logistic regression analysis confirmed the TyG-WHtR index as an independent risk factor for AMI-HF. Additionally, the TyG-WHtR index demonstrated high diagnostic efficacy in predicting HF in AMI patients, suggesting its potential as a significant predictive marker for AMI-HF.

Heart failure is the strongest predictor of mortality in patients with AMI (11). Heart failure following acute myocardial infarction increases the risk of death by 3- to 4-fold (12). The treatment of heart failure after AMI is challenging, with symptom severity ranging from mild heart failure to cardiogenic shock. Therefore, the clinical management of AMI-HF requires early intervention through effective therapeutic strategies to reduce the incidence of heart failure.

Insulin resistance disrupts myocardial energy metabolism, inhibits cellular glucose utilization, and causes fatty acids to constitute the sole energy source. As insulin resistance continues intensifies, the heart's capacity to utilize fatty acids progressively decreases, leading to increased lipid accumulation. Abnormal myocardial energy metabolism induces mitochondrial dysfunction and stimulates the excessive production of reactive oxygen species (ROS), which further impairs fatty acid oxidation capacity. This exacerbates mitochondrial dysfunction and apoptosis, resulting in lipid accumulation and cardiac fibrosis (13). These processes ultimately cause myocardial hypertrophy and contractile dysfunction, aggravate left ventricular remodeling, and contribute to the development of heart failure (14).

In recent years, the TyG index has been widely used because of its statistical simplicity, relatively low time and cost, making it suitable for large-scale population screening. Numerous high-quality clinical studies have confirmed the role of the TyG index in various diseases (15). The TyG-WHtR, which combines the waist‒to-height ratio, is more effective in reflecting central obesity and has advantages over the TyG index. A recent study analyzing 11,937 adults from the National Health and Nutrition Examination Survey (NHANES) revealed that the TyG-WHtR index had the highest predictive power for CVD mortality, significantly outperforming the TyG index (9).

A prospective cohort study (Kailuan Study) involving 8,031 participants with up to 15 years of follow-up found that the TyG-WHtR index was significantly superior to the TyG index in predicting atherosclerotic cardiovascular disease (ASCVD) risk (10). Another study based on the China Health and Retirement Longitudinal Study (CHARLS), which included 3,312 participants, confirmed through ROC curve analysis that the cumulative TyG-WHtR (AUC 0.596) demonstrated the highest diagnostic efficacy for cardiovascular disease, significantly outperforming both WHtR (AUC 0.584) and TyG index (AUC 0.568) (16). These findings demonstrate that the TyG-WHtR could serve as a more effective tool for risk stratification and identifying high-risk populations in the early stages of CVD. However, the association between the TyG-WHtR index and heart failure following acute myocardial infarction (AMI) remains unexplored.

A study analyzing 13,825 patients from the NHANES database revealed that the TyG index had an AUC of 0.602 in predicting heart failure (17). In contrast, in this study, we conducted ROC curve analysis on 738 patients and demonstrated that the TyG-WHtR index yielded a greater AUC of 0.769 for the prediction of HF. The advantages of the TyG-WHtR index include its ability to integrate metabolic indicators (TyG) with abdominal obesity parameters (WHtR), thereby more accurately reflecting the synergistic effect of metabolic obesity on cardiovascular risk. Therefore, reducing obesity and improving metabolism through physical exercise, a healthy diet, and lifestyle modifications have contributed to alleviating the substantial burden of heart failure.

Nevertheless, several limitations should be considered when these findings are interpreted. First, this was a single-center, retrospective design from a single tertiary hospital in Wuhan, which may limit generalizability. Second, sample size of 738 (136 events) yields adequate but modest statistical power, particularly for subgroup analyses. Finally, lifestyle factors and medication adherence, which could influence heart failure incidence, were not incorporated.

## 5 Conclusion

In conclusion, this study identified the TyG-WHtR index as an independent risk factor for AMI-HF. The TyG-WHtR was positively associated with the risk of AMI-HF. These findings provide theoretical support for preventing heart failure in AMI patients.

## Data Availability

The original contributions presented in the study are included in the article/Supplementary Material, further inquiries can be directed to the corresponding authors.
